# 2-Hydroxyoleic acid-inserted liposomes as a multifunctional carrier of anticancer drugs

**DOI:** 10.1080/10717544.2017.1388452

**Published:** 2017-10-13

**Authors:** Eun-Ji Jang, Woo Rim Choi, Soo-Yeon Kim, Soon-Seok Hong, Inmoo Rhee, Sang-Jin Lee, Sung Weon Choi, Han-Gon Choi, Soo-Jeong Lim

**Affiliations:** a Department of Bioscience and Biotechnology, Sejong University, Seoul, Republic of Korea;; b Immunotherapeutics Branch, Research Institute, National Cancer Center, Goyang-si, Gyeonggi-do, Republic of Korea;; c Oral Oncology Clinic, Research Institute & Hospital, National Cancer Center, Goyang-si, Gyeonggi-do, Republic of Korea;; d College of Pharmacy & Institute of Pharmaceutical Science and Technology, Hanyang University, Ansan, Republic of Korea

**Keywords:** 2-Hydroxyoleic acid, liposome, multifunctional, carrier, hydrophobic drug

## Abstract

Studies have shown that insertion of oleic acid into lipid bilayers can modulate the membrane properties of liposomes so as to improve their function as drug carriers. Considering that 2-hydroxyoleic acid (2OHOA), a potential antitumor agent currently undergoing clinical trials, is a derivative of oleic acid, we explored the possibility of developing 2OHOA-inserted liposomes as a multifunctional carrier of antitumor drugs in the present study. The insertion of 2OHOA into lipid bilayers was confirmed by surface charge determination and differential scanning calorimetry. 2OHOA insertion greatly decreased the order of dimyristoylphosphatidylcholine packing, produced a nanosized (<100 nm) dispersion, and improved the colloidal stability of liposomes during storage. Moreover, 2OHOA-inserted liposome forms exhibited greater growth inhibitory activity against cancer cells compared with free 2OHOA, and the growth-inhibitory activity of liposomal 2OHOA was selective for tumor cells. 2OHOA insertion greatly increased the liposome-incorporated concentration of hydrophobic model drugs, including mitoxantrone, paclitaxel, and all-trans retinoic acid (ATRA). The *in vitro* anticancer activity of ATRA-incorporated/2OHOA-inserted liposomes was significantly higher than that of ATRA-incorporated conventional liposomes. In a B16-F10 melanoma syngeneic mouse model, the tumor growth rate was significantly delayed in mice treated with ATRA-incorporated/2OHOA-inserted liposomes compared with that in the control group. Immunohistochemical analyses revealed that the enhanced antitumor activity of ATRA-incorporated/2OHOA-inserted liposomes was due, at least in part, to increased induction of apoptosis. Collectively, our findings indicate that 2OHOA-inserted liposomes exhibit multiple advantages as antitumor drug carriers, including the ability to simultaneously deliver two anticancer drugs – 2OHOA and incorporated drug – to the tumor tissue.

## Introduction

2-Hydroxyoleic acid (2OHOA), a synthetic derivative of oleic acid containing a hydroxyl group on the α-carbon, induces cell cycle arrest, cellular differentiation, apoptosis, and autophagy in a wide range of human cancer cells, including colon, glioma, leukemia, and breast cancer cells (Llado et al., 2010; Teres et al., 2012; Escriba et al., 2015). Based on its potent anticancer activity and low toxicity, 2OHOA has been recently designated an orphan drug for the treatment of glioma by the European Medicines Agency (Martin et al., 2013).

Cancer cells, with their uncontrolled proliferation, have very low levels of membrane sphingomyelin (SM) compared with normal cells, and this difference in SM levels, in turn, initiates a switch that triggers binding of several proliferation-promoting proteins to the membrane, thereby propagating intracellular signals that promotes the growth of cancer cells (Megha & London, 2004; Ibarguren et al., 2014). 2OHOA treatment activates sphingomyelin synthase (SMS), an enzyme that catalyzes the formation of SM, restoring membrane SM levels in cancer cells to those found in normal cells (Barcelo-Coblijn et al., 2011). In addition, 2OHOA itself is inserted into membrane phospholipids, causing alterations in membrane lipid composition and structure that change membrane protein-associated signaling and promote cell death (Martin et al., 2013). Collectively, the molecular and cellular mechanisms underlying the anticancer effects of 2OHOA appear to involve 2OHOA-induced membrane lipid remodeling in cancer cells, but not in normal cells (Escriba et al., 2015).

Liposomes have attracted research attention as drug carriers owing to their versatile ability to encapsulate hydrophilic drugs within their interior aqueous compartment and incorporate hydrophobic drugs within the hydrophobic region of the bilayer (Kim et al., 2017). Incorporation of drugs into liposomes can improve their poor aqueous solubility, limited cellular entry, and *in vivo* pharmacokinetics and tissue distribution. These favorable properties, together with the biocompatibility of phospholipids, make liposomal incorporation a promising approach for providing an administrable dosage form that improves clinical performance of drugs (Park et al., 2016).

Liposomes consist of phospholipid bilayer membranes surrounding an aqueous interior compartment. Inserting other components between phospholipid molecules is an effective approach for modulating the various properties of liposomes as drug carriers. Insertion of cholesterol inhibits the leakage of incorporated drugs from liposomes and extends the circulation time of liposomes in the blood (Senior & Gregoriadis, 1982; Deniz et al., 2010). Charged lipids improve dispersion stability by providing an electrostatic repulsion that inhibits particle aggregation. Triglyceride incorporation increases the membrane fluidity and lamellarity of liposomes composed of saturated phosphatidylcholine (PC), thereby improving their hydrophobic drug–incorporation capacity (Hong et al., 2015). Insertion of short-chain sphingolipids in the liposomal bilayer improves intracellular drug delivery to tumor cells through membrane permeabilization (Cordeiro Pedrosa et al., 2015). Bile salt enrichment in liposomal bilayers increases liposomal stability in the gastrointestinal tract and enhances the oral absorption of incorporated drugs (Niu et al., 2014). Fatty acid oleic acid incorporation decreases the order and increases the fluidity of liposomal membranes through intercalation between PC molecules; the kinked structure of oleic acid’s hydrocarbon residue intensifies this effect (Notman et al., 2007). It also increases the deformability and elasticity of liposomes, thereby enhancing the transdermal delivery of incorporated drugs (Srisuk et al., 2012).

Molecular dynamics simulation studies by Cerezo et al., undertaken to identify the interaction of PC bilayers with oleic acid or 2OHOA, showed that the interaction of these two compounds with bilayers is similar (Cerezo et al., 2011). Other experimental studies have also shown that 2OHOA, like oleic acid, interacts with and increases the fluidity of model membranes (Piotto et al., 2014). With this in mind, we hypothesized that the use of 2OHOA as a component of liposomal bilayers would modify membrane properties similar to oleic acid, leading to improved drug carrier properties of liposomes.

Research on co-treatment with two or more different anticancer agents has received considerable recent attention (Choi et al., 2017). Combination treatment may enhance the therapeutic efficacy of each agent alone and/or enable the use of reduced doses of each agent, either of which may be toxic at its usual dose. Furthermore, research indicates that by permitting simultaneous tissue and/or cellular accumulation through the enhanced permeability and retention (EPR) effect, co-delivery of two or more drugs simultaneously incorporated in nanosized carriers may offer better therapeutic efficacy than simple co-administration therapy in cancer patients (Hu et al., 2016). These observations suggest that co-delivery of 2OHOA and other chemotherapeutic drug may be a promising approach for treating cancer effectively.

High-efficiency drug incorporation is a critical factor for accelerating the clinical development of liposomal formulation; it is particularly challenging when two or more drugs are incorporated simultaneously since they may compete for the same space within liposomes. The fact that 2OHOA can be incorporated in liposomes at ∼100% efficiency by virtue of the fact that it is a constituent of liposomal membranes alleviates this difficulty. Given these many possible advantages of 2OHOA incorporation into liposomes, we undertook the present study to explore the potential usefulness of 2OHOA-inserted liposomes as multifunctional anticancer drug carriers. Here, we demonstrate that 2OHOA insertion increased membrane fluidity, hydrophobic-drug–incorporation capacity and dispersion stability of liposomes, and enhanced the antitumor efficacy of 2OHOA itself. In addition, a proof-of-concept study using 2OHOA-inserted liposomes as carriers that simultaneously deliver two drugs – all-trans retinoic acid (ATRA) and 2OHOA – demonstrates the great potential of 2OHOA-inserted liposomes as an effective antitumor platform formulation.

## Materials and methods

### Materials

2-Hydroxyoleic acid (sodium salt, 2OHOA), 1,2-dimyristoyl-sn-glycero-3-phosphocholine (DMPC), 1,2-dipalmitoyl-sn-glycero-3-phosphocholine (DPPC), and 1,2-dioleoyl-sn-glycero-3-phosphocholine (DOPC) were purchased from Avanti Polar Lipid Inc. (Alabaster, AL). Oleic acid and ATRA were purchased from Sigma-Aldrich Inc. (St. Louis, MO). Mitoxantrone dihydrochloride was bought from Tocris Bioscience Inc. (Bristol, UK). 3-(4,5-dimethylthiazol-2-yl)-2,5-diphenyltetrazolium bromide (MTT) was obtained from Amresco (Solon, OH). Paclitaxel was purchased from LC Laboratories (Boston, MA). All other chemicals were of reagent grade and used without further purification.

### Cell line and culture

UM-SCC-1 human head and neck cancer cells were kindly provided from Dr. Jeffrey N. Myers in MD Anderson Cancer Center (Zhao et al., 2011). MRC5 human fibroblast cells were purchased from the American Type Culture Collection (ATCC, Manassas, VA). Mouse B16-F10 melanoma cells were purchased from the Korea Cell Line Bank (Seoul, Republic of Korea). All cells were maintained in Dulbecco’s Modified Eagle’s Medium (Welgene, Daegu, Korea) supplemented with 10% heat-inactivated fetal bovine serum (Gibco, NY) and 100 units/ml of each of penicillin and streptomycin. Cells were grown in incubators in a humid atmosphere of 95% air and 5% CO_2_ at 37 °C.

### Preparation of liposomes

Liposomes were prepared as described in our earlier studies (Hong et al., 2016). Briefly, appropriate amounts of phosphatidylcholine and 2OHOA were first dissolved and mixed in tertiary butyl alcohol. When drug incorporation is required, drugs were dissolved together with the lipid mixture (for paclitaxel and ATRA) or first dissolved in a small volume of distilled water and then added to the tertiary butyl alcohol solution containing lipid mixture (for mitoxantrone). Mixtures were frozen at −70 °C in a deep freezer for 2 h, followed by freeze-drying in a freeze dryer (EYELA FDU-1200, Tokyo, Japan). Obtained lipid cakes were hydrated with 1 ml of 5% dextrose, briefly vortexed and then subjected to bath sonication for 30 min at temperature above the known phase transition temperature by using an ultrasonic cleaning bath (3510 R-DTH, Bransonic, Danbury, CT). To obtain a liposomal dispersion with improved homogeneity, additional sonication for 7-min was performed by using a cell disruptor (Bioruptor®, UCD-200 T, Cosmo Bio, Tokyo, Japan) set a 250 watts. The resulting liposomes were stored at 4 °C until use.

### Determination of drug concentration incorporated in liposomes

Unincorporated drugs were separated from liposomes prior to measuring the incorporated drug concentration. Unincorporated/free mitoxantrone was separated from liposomes by dialysis against 5% dextrose solution. Since our preliminary experiments showed that either paclitaxel or ATRA was practically insoluble in 5% dextrose solution, unincorporated/precipitated paclitaxel or ATRA was separated from liposomes by immediate filtering of liposomal dispersions through 0.8 μm syringe membrane filter as shown in our earlier studies (Hong et al., 2015, 2016).

After separating unincorporated drugs from liposomes, the concentration of mitoxantrone and ATRA incorporated in liposomes were determined by measuring absorbance at 660 nm (for mitoxantrone) and 340 nm (for ATRA) using a UV/VIS spectrophotometer (DU730, Beckman Coulter, FL) after disrupting the separated liposomes with 50–250-fold volume of ethanol. Standard curves pre-constructed with serial dilutions of drugs were used for the conversion of absorbance to drug concentration. For determining the incorporated paclitaxel concentration, HPLC analysis was performed as described in our earlier studies (Lee et al., 2014). Briefly, 20 μl of liposome dispersions containing paclitaxel were freeze-dried and dissolved in 200 μl of methanol and centrifuged at 13,200 rpm for 10 min. The supernatant paclitaxel samples were determined by using Nanospace SI-2 HPLC system (Shiseido Co., Ltd, Tokyo, Japan) that comprised a mobile phase delivery pump (SP 3201) and UV–visible detector (SP 3002). An Capcellpak C18 column (UG120, Shiseido, Tokyo, Japan) was used to perform the isocratic elution with a mobile phase consisting of acetonitrile and distilled water (65:35, v/v), the flow rate of 1 ml/min, and column temperature of 40 °C. A 20-μl sample was injected for each analysis, and the UV absorbance was measured at a wavelength of 227 nm.

### Physicochemical characterization of liposomes

The mean particle size and polydispersity index of liposome dispersions were measured by the dynamic light scattering method using a fiber-optic droplet analyzer (FRAP-1000, Otsuka Electronics, Osaka, Japan). Prior to measurement, liposomes were appropriately diluted with 5% dextrose solution. System was used in the auto-measuring mode. Zeta potentials, the electrical potential at the shear plane of the liposomal droplet, were determined using zetasizer (Zetasizer Nano ZSP, Malvern Instruments, Northampton, MA). Prior to measurement, liposomes were appropriately diluted with deionized water. Default instrument settings and automatic analysis were used for all measurements. Each measurement was carried out in triplicate.

The thermal behavior of the liposomal membrane was determined with Differential Scanning Calorimetry (DSC; Q2000 differential scanning calorimeter, TA Instruments, New Castle, DE). About 5 mg of the samples was taken in a standard aluminum pan and heated from 10 °C to 70 °C at a constant rate of 10 °C per minute under nitrogen atmosphere.

The size and the morphology of liposomes were also examined by negative stain transmission electron microscopy (TEM). About 5 µl of liposomal dispersions were applied to carbon-coated grids that had been glow-discharged for 3 min in air, and immediately (∼5 s) negatively stained using 1% uranyl acetate. Excess stain was removed, samples were allowed to air-dry completely, and micrographs were taken using a Tecnai G2 Spirit (FEI, Boston, MA).

The stability of the liposomes was assessed as changes in the mean particle sizes and the concentration of drugs retained in liposomes during storage at 27 °C. The concentration of drugs (ATRA) retained in liposomes was determined after separating the leaked/precipitated drugs by filtration through a membrane filter.

### Cell growth and viability assay

The growth and viability of cells was tested using an 3-(4,5-dimethylthiazol-2-yl)-2,5-diphenyltetrazolium bromide (MTT) assay. Cells (5000 cells/well for UM-SCC-1 cells, 7000 cells/well for MRC5 cells, and 1500 cells/well for B16-F10 cells) were seeded into 96-well plates. Starting from the next day, cells were incubated with varying treatments for a predetermined period of time. After incubation, the growth and viability of cells were determined by using MTT. The ability of cells to form formazan crystals by active mitochondrial respiration was determined by measuring the absorbance at 570 nm using a microplate reader (Bio-TEK, Winooski, VT) after dissolving the crystals in DMSO.

### *In vivo* anticancer efficacy study

Female C57BL/6 mouse, aged 5-weeks, were obtained from Orient Bio (Seongnam, Korea). All animal studies were performed following the guidelines approved by the Institutional Animal Care and Use Committee (IACUC) of National Cancer Center Research Institute.

B16-F10 cells (2 × 10^6 ^cells/100 μl) were inoculated subcutaneously in the dorsal area of mouse. When the tumors were palpable, the tumor mass was harvested, washed with PBS containing 1% penicillin/streptomycin and chopped into small pieces. Tumor pieces were incubated with serum-free media supplemented with 0.075% collagenase (Type IA, Sigma-Aldrich Inc. , St. Louis, MO) at 37 °C for 1 h to induce single cell suspension. After collecting the dissociated tumor cells by centrifugation, cells were seeded in collagen-coated plate in fresh DMEM media supplemented with 10% serum. When the cells reached the exponential growth phase, they were harvested and 2 × 10^6 ^cells were injected subcutaneously in the dorsal area of each mice. Tumor formation in mice was monitored and when the tumor volume reached around 15–20 mm^3^, mice were randomly divided into four treatment groups: group 1 for the empty conventional liposome control, group 2 for 2OHOA solution (200 mg/kg based on 2OHOA concentration), group 3 for ATRA-incorporated conventional liposome (10 mg/kg based on ATRA concentration), ad group 4 for ATRA-incorporated/2OHOA-inserted liposome (10 mg/kg based on ATRA concentration). Each treatment was given to mice intraperitoneally to a total of three administrations (days 0, 2, and 5). Tumor volume and body weight were measured at the scheduled time points. Tumor volume was determined using the equation (*L* ×* W*^2^)/2, where *L* represents the longest diameter and *W* represents the shortest diameter perpendicular to length.

At 8-d post-treatment, the mice were sacrificed and tumor tissues were processed for immunohistochemical analysis. Tumors were fixed in 4% paraformaldehyde in PBS, embedded in paraffin and cut into 4 μm sections. Sections were dried for 1 h at 56 °C and immunohistochemical staining was performed with the automated instrument Discovery XT (Ventana Medical Systems, Tucson, AZ) as follows: sections were deparaffinized, rehydrated with EZ prep (Ventana Medical Systems, Tucson, AZ), and washed with reaction buffer (Ventana Medical Systems, Tucson, AZ). The antigens were retrieved with heat treatment in Tris–EDTA buffer (CC1, Ventana Medical Systems, Tucson, AZ) at 90 °C for 30 min with anti-cleaved caspase 3 antibodies (Cell Signaling Technology, Danverse, MA).

### Statistical analysis

Statistically significant differences between values obtained in different compositions of liposomes or under different experimental conditions were determined using two-tailed unpaired Student’s *t*-tests or one-way analysis of variance (ANOVA).

## Results and discussion

### Effects of 2OHOA incorporation on the physicochemical properties of liposomes

**Figure 1.**
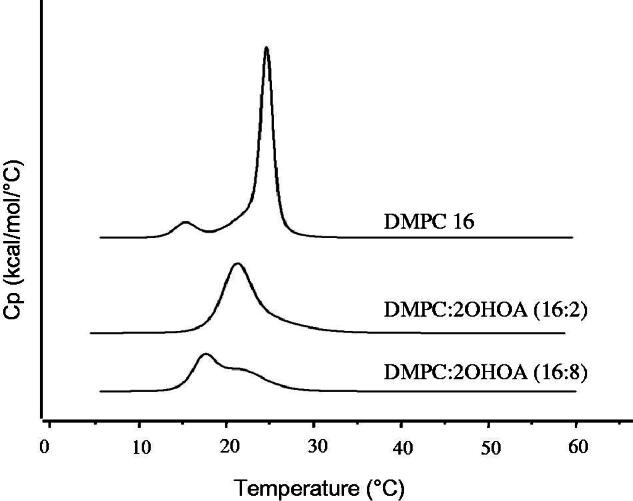
Effect of 2OHOA content on DSC thermograms of DMPC liposomes. Liposomes were prepared with the indicated molar ratio of DMPC and 2OHOA.

Alterations in the packing of PC molecules within the bilayer are known to affect the thermodynamics of the lipid phase transition. To investigate whether the packing of PC within the liposomal bilayer was altered when liposomes were prepared in the presence of 2OHOA, we performed DSC studies with DMPC liposomes prepared with or without 2OHOA supplementation. Heating thermograms shown in Figure 1 indicate the presence of two transition peaks, a smaller one at 14.95 °C and a more intense one at 24.3 °C, in liposomes prepared with DMPC alone. These two peaks correspond to the known pre-transition and major transition temperature (*T*_m_) of DMPC, respectively (Needham et al., 1988; Wrobel et al., 2011). Pre-transition is a low-enthalpy transition reflecting the membrane’s change from the gel phase to the rippled gel phase, and the main phase transition is associated with the change of membranes from the rigid gel phase to the fluid liquid crystalline phase. The preparation of DMPC liposomes by supplementation with 11% 2OHOA had a major influence on both transition peaks: the pre-transition peak completely disappeared and the main transition peak was shifted to a lower temperature (21.9 °C) and broadened with a decreased enthalpy. Elimination of the pre-transition peak implies that the added compound, 2OHOA, interacts with the head group of DMPC (Barry et al., 2009). Our data are in agreement with the assumption made by molecular simulations that the polar heads of 2OHOA locate slightly below the polar head group of the phospholipid (Cerezo et al., 2011). The *T*_m_ shift to a lower temperature indicates that 2OHOA was successfully inserted into the PC bilayers of liposomes, thereby bringing the bilayer from an ordered gel to a disordered fluid state. Studies by others have demonstrated a similar decrease in the lateral forces between PC head groups (Notman et al., 2007) and disruption of the ordered packing of the phospholipid molecules by oleic acid. The *cis* double bond of oleic acid molecules causes a kinked lipid tail, disrupting membrane order and lowering the *T*_m_ (Srisuk et al., 2012). Taken together, these data suggest that 2OHOA is intercalated between PC molecules when added together with PC during the preparation of liposomes and thus increases the fluidity of the membrane.

Further increases in the 2OHOA content up to 33% caused greater broadening and splitting of the main transition peak (Figure 1). This effect may be attributable to the heterogeneous distribution of 2OHOA when present at high content in the lipid bilayer, as reported in the case of a high content of oleic acid, which causes phase separation with possible pooling of oleic acid within the bilayer (Cerezo et al., 2011; Prades et al., 2012).

**Figure 2.**
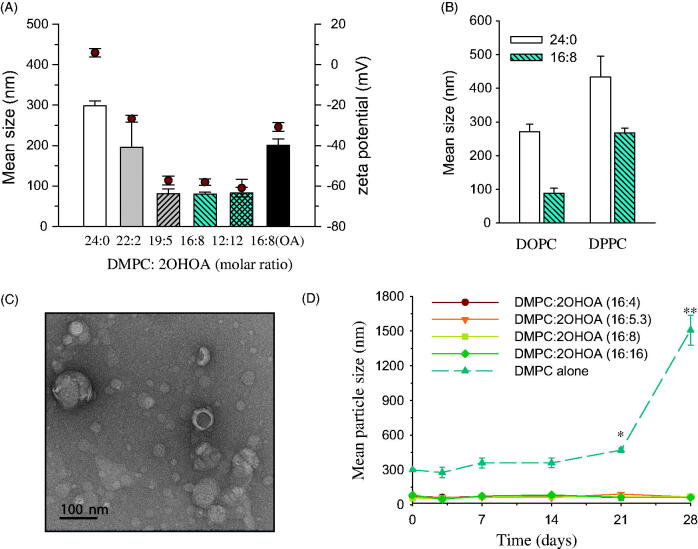
Effects of 2OHOA insertion on the physicochemical characteristics of liposomes. (A) Effects of 2OHOA content on the mean particle size and zeta potential of liposomes. Liposomes were prepared with the indicated molar ratio of DMPC and 2OHOA. For comparison, oleic acid-inserted liposomes were prepared with a 16:8 molar ratio of DMPC and oleic acid. (B) Effect of 2OHOA insertion on the mean size of liposomes prepared with DOPC or DPPC. Liposomes were prepared with a 16:8 molar ratio of PC and 2OHOA. (C) Representative TEM image of 2OHOA-inserted liposomes. Liposomes were prepared with a 16:8 molar ratio of DMPC:2OHOA. (D) Time-dependent changes in the mean particle size of liposomes with varying 2OHOA content. Liposomes prepared with the indicated ratio of DMPC and 2OHOA were stored at room temperature for up to 28 days. Data are expressed as means ± SD (*n* = 3; *p < .05, **p < .005 compared with the initial condition).

We next investigated the effect of 2OHOA content on the mean particle size and surface charge of liposomes. The mean size of DMPC liposomes tended to decrease in a 2OHOA content-dependent manner; the mean size of liposomes composed of DMPC alone was the largest (∼300 nm) and was reduced to <100 nm when liposomes were prepared with ≥20.8% 2OHOA (Figure 2(A)). These data indicate that, in terms of particle size, 2OHOA incorporation would enable increased tumor accumulation of DMPC liposomes through the EPR effect, which allows particles in the range of 10–100 nm to accumulate relatively easily in tumor sites through vascular fenestrations (Maeda et al., 2000). In accord with our data, a significant decrease in liposome size to below 100 nm by oleic acid incorporation has been reported in other studies (Huang et al., 2011; Srisuk et al., 2012). The polydispersity index of 2OHOA-inserted liposomes ranged between 0.2 and 0.35, indicating a size homogeneity of 2OHOA-inserted liposomes (data not shown). 2OHOA incorporation into liposomes prepared with either DOPC or DPPC also greatly reduced the mean size of liposomes, implying that 2OHOA increased the colloidal stability of liposomes regardless of PC type (Figure 2(B)).

Zeta potential is a measure of the surface charge of colloidal particles. Liposomes composed of DMPC alone exhibited zeta potential values close to zero. In contrast, the surface charge of 2OHOA-inserted liposomes showed a tendency toward increased negativity with increases in 2OHOA content (Figure 2(A)). It seems likely that the sodium salt form of 2OHOA used in the present study was present in the liposomal bilayer as a charged form, thereby contributing to the negativity of the liposome surface charge. In general, liposomes with high zeta potential (>50 mV negative or positive) are considered physically stable owing to the electric repulsion between particles; thus, our data indicate that DMPC liposomes can be stabilized electrostatically by incorporation of ≥20.8% 2OHOA. Compared with liposomes composed of DMPC alone, the surface charge of liposomes containing 33% oleic acid became more negative, but the negativity was much less than that for liposomes containing the same 2OHOA content. Part of oleic acid is probably present as a deprotonated form (oleate) under these experimental conditions, and thus the surface charge of liposomal oleic acid becomes negative, as reported in earlier studies (Huang et al., 2011).

A representative TEM image of 2OHOA-inserted liposomes revealed mostly spherical vehicles in the size range of 10–100 nm (Figure 2(C)). It is well known that the mean particle diameter estimated by TEM analysis tends to be smaller than the hydrodynamic diameter determined by the dynamic light scattering (DLS) method. To assess the 2OHOA effect on the stability of DMPC liposomes, we compared time-dependent changes in the mean particle size during storage among liposomes containing varying 2OHOA content. During storage at 27 °C, liposomes composed of DMPC alone exhibited a gradual increase in mean particle size up to 3 weeks, and then showed a sharp increase (>5-fold compared with the initial size), implying aggregation of electrically neutral DMPC liposomes during storage. In contrast, no significant changes in the mean size of 2OHOA-inserted liposomes were observed regardless of 2OHOA content during 4-week storage at 27 °C (Figure 2(D)). Collectively, these data indicate that 2OHOA is inserted into the lipid bilayer and increases its fluidity in a manner similar to that of oleic acid, and this incorporation of 2OHOA improves the stability of liposomes.

### Effect of 2OHOA incorporation on the antitumor activity of 2OHOA

**Figure 3.**
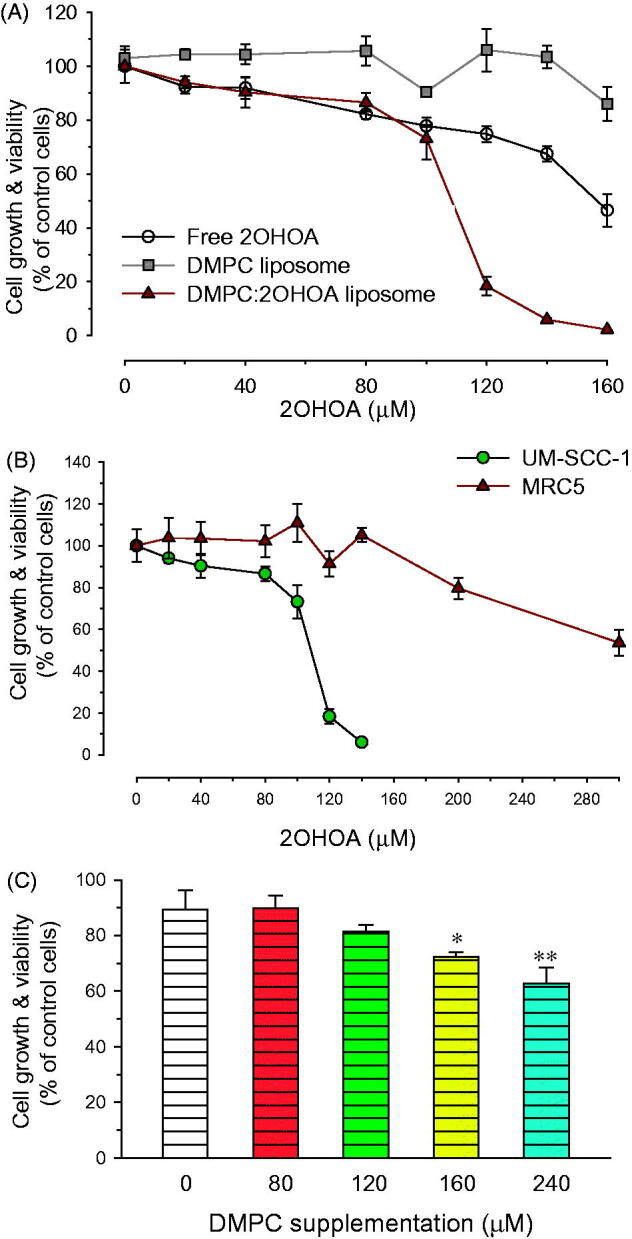
Effects of liposomal insertion on the anticancer activity of 2OHOA. (A) Comparison of the anticancer efficacy of free and liposome-inserted 2OHOA. Cell growth and viability were determined by MTT assays after incubating UM-SCC-1 tumor cells with different formulations for 72 h. The free 2OHOA solution was prepared by dissolving 2OHOA in DMSO, and liposomes were prepared with DMPC alone or a 16:8 molar ratio of DMPC:2OHOA. Liposomes composed of DMPC alone were added to cells after preparing dilutions corresponding to the dilution of DMPC:2OHOA liposomes. (B) Effects of liposome-inserted 2OHOA on the growth and viability of cancer cells and normal cells. Cancer cells (UM-SCC-1) and normal cells (MRC5) were incubated with different dilutions of DMPC:2OHOA (molar ratio, 16:8) liposomes for 72 h, after which MTT assays were performed. (C) Effect of DMPC supplementation on the anticancer activity of 2OHOA. UM-SCC1 cells were incubated with 120 μM 2OHOA (DMSO solution) in the presence of the indicated concentration of DMPC (DMSO solution) for 48 h, after which MTT assays were performed. Results are expressed as percentage growth (means ± S.D. of triplicate wells) relative to control cells (*p < .05, **p < .005 compared with 2OHOA treatment alone).

Treatment of UM-SCC-1 tumor cells with 2OHOA inhibited the growth of tumor cells in a concentration-dependent manner. Of the free and liposome-inserted form of 2OHOA, the liposome-inserted form exhibited more potent growth inhibitory activity: 120 μM 2OHOA inserted into liposomes inhibited cellular proliferation by 81.7% ± 3.6%, whereas the same concentration of free 2OHOA inhibited proliferation by only 25.2% ± 2.9%. The 50% inhibitory concentration (IC_50_) of free 2OHOA was approximately 1.5-fold higher than that of liposomal 2OHOA, indicating that incorporation of 2OHOA increased the antitumor activity of liposomes (Figure 3(A)). Liposomes composed of DMPC alone did not significantly inhibit the growth of cells within the concentration range used in this study, indicating that the antitumor effect of liposome treatment was mediated by the 2OHOA inserted into the liposomal bilayer.

2OHOA has been shown to exert growth inhibitory effects in tumor cells, but not in normal cells (Martin et al., 2013). Since liposomal incorporation enhanced the growth-inhibitory effect of 2OHOA on tumor cells, it was of interest to determine whether liposomal incorporation affected the selectivity of 2OHOA for inhibiting growth of tumor cells. As shown in Figure 3(B), incubation with liposomal 2OHOA for 72 h slightly inhibited the proliferation of non-tumor MRC5 cells only at the ≥200 μM concentration of 2OHOA, implying that the selectivity of 2OHOA for tumor cells is retained after liposomal incorporation.

To better understand why liposomal 2OHOA exhibits more potent antitumor activity than free 2OHOA, we performed separate experiments in which UM-SCC-1 cells were treated with a fixed concentration of free 2OHOA in the presence of varying concentrations of free DMPC. The growth-inhibitory effect of 2OHOA towards UM-SCC-1 cells increased with increasing supplemented DMPC concentration, implying that combined treatment with DMPC enhances the effect of 2OHOA on tumor cell growth (Figure 3(C)). Considering that 2OHOA-activated SMase activated catalyzes the synthesis of SM through transfer of the phosphatidyl head group of PC onto the primary hydroxyl of ceramide, DMPC supplementation may increase the available concentration of substrate (PC) in the vicinity of SMase, thereby facilitating SM synthesis. Differences in the mechanism of free and liposomal 2OHOA interactions with the cell membrane may be another reason causing the differences in the antitumor potency: liposomal 2OHOA fuses with the membrane, whereas free 2OHOA diffuses into the membranes on an individual molecule basis. In this context, Huang et al. demonstrated that liposomal oleic acid kills bacteria more potently than free OA, because liposomal oleic acid fuses with the bacterial membrane more rapidly than free OA (Huang et al., 2011).

### Effects of 2OHOA insertion on the hydrophobic-drug–incorporation capacity of liposomes

**Figure 4.**
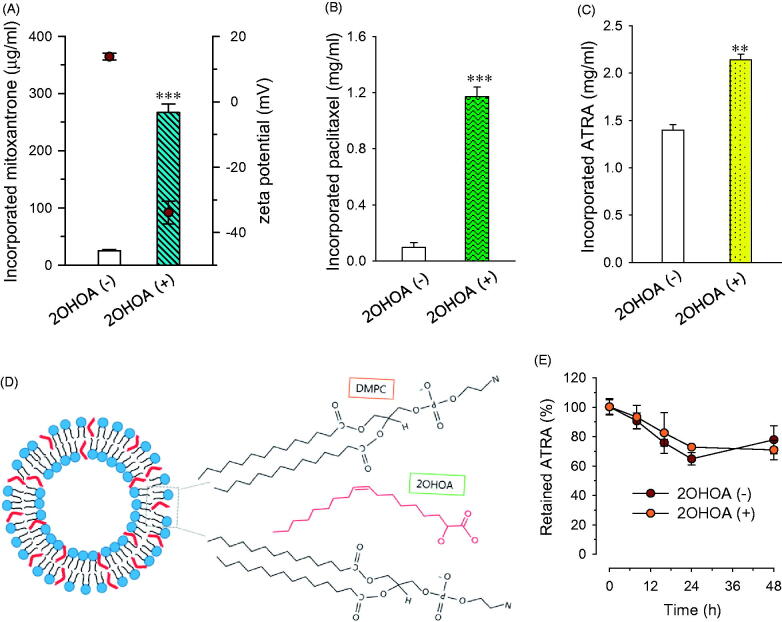
Effects of 2OHOA insertion on the drug-incorporation capacity of liposomes. Effects of 2OHOA insertion on the liposome-incorporated concentration of (A) mitoxantrone, (B) paclitaxel, and (C) ATRA. (A and B) Liposomes were prepared with DMPC alone (24 μmole/ml) or DMPC:2OHOA (molar ratio, 16:8; total, 24 μmole/ml) together with 0.25 mg of mitoxantrone or 1.5 mg of paclitaxel. (C) Liposomes were prepared with DMPC:CHOL (molar ratio, 24:2; total, 26 μmole/ml) or DMPC:2OHOA:CHOL (molar ratio, 12:12:2; total, 26 μmole/ml) containing 2 mg of ATRA. Data are expressed as means ± SD (*n* = 3; **p < .005, ***p < .0001 compared with 2OHOA-free liposomes). (D) Schematic depiction of the structure of 2OHOA-inserted liposomes. (E) Effect of 2OHOA insertion on the time-dependent leakage of ATRA incorporated in liposomes. Liposomes were prepared as described in (C) and stored at 27 °C up to 48 h.

Incorporation of hydrophobic drugs through embedding within the lipid bilayer has been shown to overcome the solubility limitations of drugs, offering an effective means for providing injectable formulations. High drug-incorporation capacity, however, is required for the clinical development of liposomes in a cost-effective dosage form. To investigate 2OHOA effects on the drug-incorporation capacity of liposomes, we chose three hydrophobic anticancer drugs in clinical use: mitoxantrone, paclitaxel, and ATRA (Shapira et al., 2010; Hong et al., 2016; Silva et al., 2016). Incorporation of 33% 2OHOA improved the incorporation of all three drugs, increasing the concentrations of mitoxantrone, paclitaxel, and ATRA by 10.7-, 13.5- and 1.5-folds, respectively (Figure 4(A–C)). It is conceivable that the intercalation of 2OHOA between DMPC molecules decreased the ordering of lipid acyl chains and thus facilitated the incorporation of drugs during the preparation of liposomes. In addition, the kinked structure of 2OHOA may also contribute to providing wider spaces for accommodating hydrophobic drugs, as depicted in Figure 4(D).

Among the three drugs, the effect of 2OHOA on drug-incorporation capacity was greater for paclitaxel and mitoxantrone than ATRA, possibly reflecting differences in the molecular structures of these drugs. Literatures have shown that incorporation of paclitaxel into liposomal membranes composed of saturated PC is particularly challenging, owing to the bulky and asymmetrical paclitaxel structure (Hong et al., 2016); thus, it seems likely that paclitaxel gains the most benefit from gap widening between PC molecules by 2OHOA. Earlier studies have demonstrated that incorporation of the cationic drug mitoxantrone into liposomes is greatly enhanced by incorporation of anionic phospholipids owing to the resulting electrostatic interactions (Li et al., 2008). In this context, it is plausible that mitoxantrone–2OHOA electrostatic interactions in our formulation, in addition to drug–PC hydrophobic interactions, contributed to the effectiveness of mitoxantrone incorporation. Indeed, the zeta potential values of mitoxantrone-incorporated liposomes indicate positive (+13.8 mV) and negative (–33.9 mV) surface charges of plain and 2OHOA-inserted liposomes, respectively (Figure 4(A)). Compared with the highly negative zeta potential value for drug-free/2OHOA-incoporated liposomes, the change in negativity of mitoxantrone-incorporated/2OHOA-inserted liposomes was much less (from –58.1 to −33.9 mV), supporting this assumption.

Effect of 2OHOA insertion on the stability of drug-loaded liposomes was evaluated by monitoring the leakage of liposome-incorporated drugs during storage at 27 °C. Leakage test was performed with ATRA-incorporated liposomes since the initial concentrations of either paclitaxel or mitoxantrone incorporated into 2OHOA-free liposomes were too low to monitor the time-dependent drug leakage. When liposomes were stored at 27 °C, ATRA was leaked from liposomes in a time-dependent manner regardless of 2OHOA incorporation but the leakage of ATRA after 48 h was less than 30% of initial incorporated ATRA in both formulations (Figure 4(E)). It indicates that the 2OHOA insertion does not exaggerate the drug leakage/precipiation from liposomes despite of higher incorporated drug concentration.

### Effects of 2OHOA insertion on the antitumor activity of ATRA *in vitro* and *in vivo*

To explore the therapeutic potential of 2OHOA-inserted liposomes as more efficient carriers of antitumor drug, we next sought to determine whether combined treatment with 2OHOA and an antitumor drug enhanced the cytotoxic effects of the latter in tumor cells. We performed these studies using B16-F10 murine melanoma cells in culture and a syngeneic mouse model, reflecting the fact that melanoma, one of the most aggressive and hardest to treat cancers, requires novel combination therapeutic strategies (Ugurel et al., 2016). ATRA, the drug of choice for the treatment of acute promyelocytic leukemia, has been shown to induce cell-growth inhibition, differentiation, and/or apoptosis in a broad spectrum of cancers, including melanoma (Niles, 2003). In addition to overcoming solubility limitations, liposomal incorporation of ATRA has been shown to improve the chemical stability and tumor accumulation of the drug (Siddikuzzaman & Grace, 2012). The multiple benefits expected to be gained by incorporation ATRA in liposomes prompted us to select ATRA as a model anticancer drug for this study.

**Figure 5.**
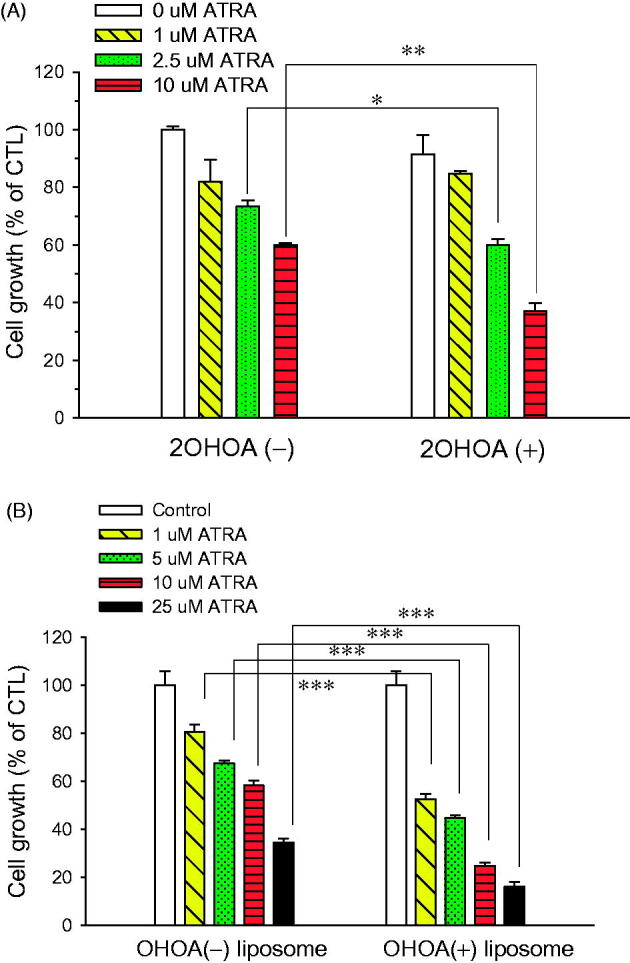
Effect of 2OHOA on the in vitro anticancer activity of ATRA. The growth-inhibitory effect of ATRA in mouse B16–F10 melanoma cells was evaluated by MTT assays after a 72-h incubation. (A) Growth-inhibitory effects of free ATRA in the presence or absence of free 2OHOA (150 μM). (B) Comparison of the growth-inhibitory effects of ATRA incorporated in conventional liposomes or 2OHOA-inserted liposomes. Liposomes used in the study were composed of DMPC:CHOL:ATRA (molar ratio, 24:2:0.53) and DMPC:2OHOA:CHOL:ATRA (molar ratio, 12:12:2:0.53). Data are expressed as percentage growth (means ± S.D. of triplicate wells) relative to control cells.

When cultured B16-F10 cells were incubated with ATRA in the presence or absence of 2OHOA for 72 h, ATRA inhibited cell growth in a concentration-dependent manner. Compared with ATRA treatment alone, combination treatment with 2OHOA inhibited cell growth to a greater extent: 2.5 and 10 μM ATRA alone decreased cell growth by 26.6% and 40.0%, respectively, whereas the corresponding values for same doses of ATRA together with 150 μM 2OHOA were 40.0% and 62.8%. At a concentration of 150 μM, 2OHOA alone decreased cell growth by less than 10% (Figure 5(A)). These data indicate that when used in combination, ATRA and 2OHOA cooperatively exert anti-proliferative effects against melanoma cells. As a result, 2OHOA cotreatment lowered the ATRA concentration that was capable of inducing cell-growth inhibition, implying potential therapeutic benefits of combined ATRA and 2OHOA treatment in melanoma.

Based on data for combination treatment *in vitro*, we prepared ATRA-incorporated liposomes with or without 2OHOA insertion. ATRA incorporated into 2OHOA-inserted liposomes inhibited cell growth more effectively than 2OHOA-free liposomes at all concentrations used: a DMPC:CHOL:ATRA liposome formulation (10 μM ATRA) decreased cell growth by 41.8%, whereas the same concentration of ATRA as a DMPC:2OHOA:CHOL:ATRA liposome formulation decreased cell growth by 75.2% (Figure 5(B)). These results indicate that the IC_50_ of ATRA was >10 μM for the 2OHOA-free liposomal formulation but <10 μM for the 2OHOA-inserted liposomal formulation, suggesting that ATRA in a 2OHOA-inserted liposome-incorporated form possesses more potent antitumor activity.

**Figure 6.**
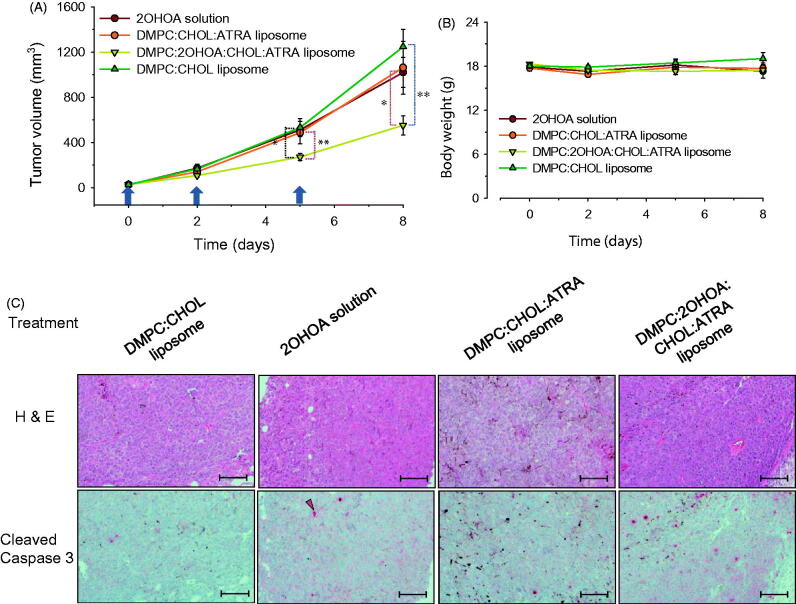
*In vivo* antitumor study. (A) Tumor growth and (B) body weight changes were monitored in a B16–F10 syngeneic mouse tumor model after treatment with free 2OHOA, ATRA-incorporated conventional liposomes, ATRA-incorporated/2OHOA-inserted liposomes, or empty conventional liposomes. Upward arrows indicate treatment time points. Data are presented as means ± SEM (*n* = 6–7; *p < .05, **p < .005). (C) Representative immunohistochemical images of syngeneic B16–F10 tumors obtained from each group of mice after treatment. Scale bars: 100 μm. The 2OHOA solution for treatment was prepared by dissolving 2OHOA at 6.4 mg/ml in distilled water containing 0.25% hydroxypropylmethylcellulose (Shin-Etsu Co., Tokyo, Japan) and 0.2% Tween-80 (Sigma-Aldrich Inc., St. Louis, MO). Empty conventional liposomes were composed of a 40:3 molar ratio of DMPC:CHOL. The composition of ATRA-incorporated conventional liposomes and ATRA-incorporated/2OHOA-inserted liposomes used were DMPC:CHOL:ATRA (molar ratio, 24:1.9:0.85) and DMPC:2OHOA:CHOL:ATRA (molar ratio, 12:12:1.9:0.78), respectively. The arrow indicates the active caspase-3 fragment; the brown-black pigments are melanin.

We next assessed the antitumor activity of ATRA incorporated in conventional or 2OHOA-inserted liposomes using a mouse model of subcutaneously implanted B16-F10 cells. Mice were divided into the following four treatment groups: empty conventional liposomes (control), free 2OHOA, ATRA-incorporated conventional liposomes, and ATRA-incorporated/2OHOA-inserted liposomes. Mice in each group were treated with the indicated formulations three times. Compared with the tumor growth in the control group treated with empty vehicle (DMPC:CHOL liposome), the growth rate of tumors in the other three mouse groups tended to be delayed. After 8 d, tumor mass volumes in mice treated with free 2OHOA, ATRA-incorporated conventional liposome, or ATRA-incorporated/2OHOA-inserted liposome groups were 18.1%, 14.7%, and 55.6% smaller, respectively, than those of controls. However, differences compared with control mice were statistically significant only for groups treated with ATRA-incorporated/2OHOA-inserted liposomes at 5 and 8 d postinjection (*p* < .05, Figure 6(A)). Tumor growth in ATRA-incorporated/2OHOA-inserted liposome groups tended to be retarded compared with that in groups treated with ATRA-incorporated conventional liposomes, but the difference fell short of statistical significance (*p* = .0512 and .0512 on days 5 and 8). Taken together, these results suggest additive effects of treatment in syngeneic tumor models, based on an assessment of tumor growth.

Body weight change was also assessed as an indicator of side effects and treatment toxicities. Tumor-bearing mice in each group showed slight or no weight gains during the experiment, but no significant weight loss was observed in any treatment groups (Figure 6(B)). These data indicate that no treatments caused severe toxicity leading to body weight loss at the doses and dosing intervals used, implying that 2OHOA-inserted liposomal formulations are safe for treating melanoma.

Immunohistochemical analyses revealed that immunoreactive cells positive for the apoptotic marker, cleaved caspase-3, were barely detectable in tumors of control mice, whereas a few positive cells were found in both 2OHOA-treated mice and DMPC:CHOL:ATRA liposome-treated mice. In contrast, tumors of DMPC:2OHOA:CHOL:ATRA liposome-treated mice had a considerably higher proportion of cleaved caspase-3-expressing cells (Figure 6(C)). These results indicate that ATRA-incorporated/2OHOA-inserted liposomes exerted higher antitumor activity compared with that of ATRA-incorporated conventional liposomes, a free 2OHOA solution, and empty conventional liposomes by increasing tumor cell apoptosis. ATRA is known to exert its antitumor activity, at least in part, through induction of either intrinsic or extrinsic apoptosis (Noy, 2010). In this context, literature reports have shown that the membrane lipid reorganization caused by 2OHOA may cause clustering of the death receptor, Fas, and initiate the extrinsic apoptosis pathway, leading to apoptotic cell death (Llado et al., 2010). Taken together, our findings indicate that ATRA-2OHOA co-delivered by liposomes to tumors may converge at some point in the apoptotic pathway leading to cell death.

## Conclusions

In the present study, we explored the potential of 2OHOA-inserted liposomes as versatile antitumor drug carriers, based on the assumption that 2OHOA modulates the membrane properties of lipid bilayers to further increase their utility as hydrophobic drug carriers, and that 2OHOA inserted into liposomes may cooperate with liposome-incorporated anticancer drugs to exert enhanced antitumor activity, reflecting the simultaneous delivery of two anticancer drugs (2OHOA and incorporated drug) to the tumor tissue. The data presented here indicate that 2OHOA inserted in liposomes decreases the packing order of PC bilayers and thus greatly improves the concentration of hydrophobic drugs incorporated into liposomes. In addition, 2OHOA insertion improved the colloidal stability of liposomes by providing electrostatic stabilization. Furthermore, the antitumor activity of 2OHOA was increased by virtue of incorporation into liposomes, and ATRA-incorporated/2OHOA-inserted liposomes exerted greater antitumor activity against melanoma *in vitro* and *in vivo*. We speculate that the more potent antitumor activity of ATRA-incorporated/2OHOA-inserted liposomes compared with ATRA-incorporated conventional liposomes is achieved through multiple benefits provided by 2OHOA insertion in liposomes, as noted above. While not investigated here, increased tumor cell uptake of ATRA incorporated in 2OHOA-inserted liposomes, through increased endocytosis and/or fusion of liposomes, compared with that of ATRA incorporated into conventional liposomes may be an additional factor that contributes to the enhanced antitumor activity. In conclusion, 2OHOA-inserted liposomes show promising potential as an effective ATRA carrier by exhibiting multiple therapeutic advantages, including the ability to simultaneously deliver two anticancer drugs – 2OHOA and ATRA – to the melanoma tissue. The potential effectiveness of 2OHOA-inserted liposomes as anticancer drug carriers may be expanded to other hydrophobic anticancer drugs. Considering that the current clinical trials of 2OHOA are aimed at patients with glioma, it would be of interest to further investigate the therapeutic efficacy of liposomes codelivering 2OHOA and drugs frequently used in glioma patients.

## References

[CIT0001] Barcelo-Coblijn G, Martin ML, De Almeida RF, et al. (2011). Sphingomyelin and sphingomyelin synthase (SMS) in the malignant transformation of glioma cells and in 2-hydroxyoleic acid therapy. Proc Natl Acad Sci USA 108:19569–74. 22106271 10.1073/pnas.1115484108 PMC3241787

[CIT0002] Barry J, Fritz M, Brender JR, et al. (2009). Determining the effects of lipophilic drugs on membrane structure by solid-state NMR spectroscopy: the case of the antioxidant curcumin. J Am Chem Soc 131:4490–8. 19256547 10.1021/ja809217u PMC2748423

[CIT0003] Cerezo J, Zuniga J, Bastida A, et al. (2011). Atomistic molecular dynamics simulations of the interactions of oleic and 2-hydroxyoleic acids with phosphatidylcholine bilayers. J Phys Chem B 115:11727–38. 21882864 10.1021/jp203498x

[CIT0004] Choi JH, Lee YJ, Kim D. (2017). Image-guided nanomedicine for cancer. J Pharm Investig 47:51–64.

[CIT0005] Cordeiro Pedrosa LR, Van Tellingen O, Soullie T, et al. (2015). Plasma membrane targeting by short chain sphingolipids inserted in liposomes improves anti-tumor activity of mitoxantrone in an orthotopic breast carcinoma xenograft model. Eur J Pharm Biopharm 94:207–19. 25982691 10.1016/j.ejpb.2015.05.003

[CIT0006] Deniz A, Sade A, Severcan F, et al. (2010). Celecoxib-loaded liposomes: effect of cholesterol on encapsulation and in vitro release characteristics. Biosci Rep 30:365–73. 19900165 10.1042/BSR20090104

[CIT0007] Escriba PV, Busquets X, Inokuchi J, et al. (2015). Membrane lipid therapy: modulation of the cell membrane composition and structure as a molecular base for drug discovery and new disease treatment. Prog Lipid Res 59:38–53. 25969421 10.1016/j.plipres.2015.04.003

[CIT0008] Hong SS, Choi JY, Kim JO, et al. (2016). Development of paclitaxel-incorporated liposomal nanocarrier stabilized by triglyceride incorporation. Int J Nanomedicine 11:4465–77. 27660440 10.2147/IJN.S113723 PMC5019274

[CIT0009] Hong SS, Kim SH, Lim SJ. (2015). Effects of triglycerides on the hydrophobic drug loading capacity of saturated phosphatidylcholine-based liposomes. Int J Pharm 483:142–50. 25667981 10.1016/j.ijpharm.2015.02.013

[CIT0010] Hu T, Cao H, Yang C, et al. (2016). LHD-modified mechanism-based liposome coencapsulation of mitoxantrone and prednisolone using novel lipid bilayer fusion for tissue-specific colocalization and synergistic antitumor effects . ACS Appl Mater Interfaces 8:6586–601. 26907854 10.1021/acsami.5b10598

[CIT0011] Huang CM, Chen CH, Pornpattananangkul D, et al. (2011). Eradication of drug resistant staphylococcus aureus by liposomal oleic acids. Biomaterials 32:214–21. 20880576 10.1016/j.biomaterials.2010.08.076 PMC2987540

[CIT0012] Ibarguren M, Lopez DJ, Escriba PV. (2014). The effect of natural and synthetic fatty acids on membrane structure, microdomain organization, cellular functions and human health. Biochim Biophys Acta 1838:1518–28. 24388951 10.1016/j.bbamem.2013.12.021

[CIT0013] Kim CH, Lee SG, Kang MJ, et al. (2017). Surface modification of lipid-based nanocarriers for cancer cell-specific drug targeting. J Pharm Investig 47:203–27.

[CIT0014] Lee EH, Hong SS, Kim SH, et al. (2014). Computed tomography-guided screening of surfactant effect on blood circulation time of emulsions: application to the design of an emulsion formulation for paclitaxel. Pharm Res 31:2022–34. 24549824 10.1007/s11095-014-1304-8

[CIT0015] Li C, Cui J, Wang C, et al. (2008). Encapsulation of mitoxantrone into pegylated SUVs enhances its antineoplastic efficacy. Eur J Pharm Biopharm 70:657–65. 18582570 10.1016/j.ejpb.2008.05.019

[CIT0016] Llado V, Gutierrez A, Martinez J, et al. (2010). Minerval induces apoptosis in Jurkat and other cancer cells. J Cell Mol Med 14:659–70. 19413889 10.1111/j.1582-4934.2008.00625.x PMC3823464

[CIT0017] Maeda H, Wu J, Sawa T, et al. (2000). Tumor vascular permeability and the EPR effect in macromolecular therapeutics: a review. J Control Release 65:271–84. 10699287 10.1016/s0168-3659(99)00248-5

[CIT0018] Martin ML, Barcelo-Coblijn G, De Almeida RF, et al. (2013). The role of membrane fatty acid remodeling in the antitumor mechanism of action of 2-hydroxyoleic acid. Biochim Biophys Acta 1828:1405–13. 23360770 10.1016/j.bbamem.2013.01.013

[CIT0019] Megha, London E. (2004). Ceramide selectively displaces cholesterol from ordered lipid domains (rafts): implications for lipid raft structure and function. J Biol Chem 279:9997–10004. 14699154 10.1074/jbc.M309992200

[CIT0020] Needham D, Mcintosh TJ, Evans E. (1988). Thermomechanical and transition properties of dimyristoylphosphatidylcholine/cholesterol bilayers. Biochemistry 27:4668–73. 3167010 10.1021/bi00413a013

[CIT0021] Niles RM. (2003). Vitamin A (retinoids) regulation of mouse melanoma growth and differentiation. J Nutr 133:282S–6S. 12514310 10.1093/jn/133.1.282S

[CIT0022] Niu M, Tan Y, Guan P, et al. (2014). Enhanced oral absorption of insulin-incorporated liposomes containing bile salts: a mechanistic study. Int J Pharm 460:119–30. 24275447 10.1016/j.ijpharm.2013.11.028

[CIT0023] Notman R, Noro MG, Anwar J. (2007). Interaction of oleic acid with dipalmitoylphosphatidylcholine (DPPC) bilayers simulated by molecular dynamics. J Phys Chem B 111:12748–55. 17939702 10.1021/jp0723564

[CIT0024] Noy N. (2010). Between death and survival: retinoic acid in regulation of apoptosis. Annu Rev Nutr 30:201–17. 20415582 10.1146/annurev.nutr.28.061807.155509

[CIT0025] Park JY, Kim M-G, Shim G, et al. (2016). Lipid-based antigen delivery systems. J Pharm Investig 46:295–304. 10.1007/s40005-016-0258-8 PMC7100357 32226640

[CIT0036] Piotto S, Concilio S, Bianchino E, et al. (2014). Differential effect of 2-hydroxyoleic acid enantiomers on protein (sphingomyelin synthase) and lipid (membrane) targets. Biochim Biophys Acta 1838:1628–37. 24412218 10.1016/j.bbamem.2013.12.023

[CIT0026] Prades J, Funari SS, Gomez-Florit M, et al. (2012). Effect of a 2-hydroxylated fatty acid on cholesterol-rich membrane domains. Mol Membr Biol 29:333–43. 22830943 10.3109/09687688.2012.705023

[CIT0027] Senior J, Gregoriadis G. (1982). Stability of small unilamellar liposomes in serum and clearance from the circulation: the effect of the phospholipid and cholesterol components. Life Sci 30:2123–36. 7109841 10.1016/0024-3205(82)90455-6

[CIT0028] Shapira A, Markman G, Assaraf YG, et al. (2010). Beta-casein-based nanovehicles for oral delivery of chemotherapeutic drugs: drug-protein interactions and mitoxantrone loading capacity. Nanomedicine 6:547–55. 20100598 10.1016/j.nano.2010.01.003

[CIT0029] Siddikuzzaman, Grace VM. (2012). Inhibition of metastatic lung cancer in C57BL/6 mice by liposome incorporated all trans retinoic acid (ATRA). Int Immunopharmacol 14:570–9. 23021983 10.1016/j.intimp.2012.09.008

[CIT0030] Silva EL, Lima FA, Carneiro G, et al. (2016). Improved in vitro antileukemic activity of all-trans retinoic acid incorporated in cholesteryl butyrate solid lipid nanoparticles. J Nanosci Nanotechnol 16:1291–300. 27433579 10.1166/jnn.2016.11677

[CIT0031] Srisuk P, Thongnopnua P, Raktanonchai U, et al. (2012). Physico-chemical characteristics of methotrexate-entrapped oleic acid-containing deformable liposomes for in vitro transepidermal delivery targeting psoriasis treatment. Int J Pharm 427:426–34. 22310459 10.1016/j.ijpharm.2012.01.045

[CIT0032] Teres S, Llado V, Higuera M, et al. (2012). 2-Hydroxyoleate, a nontoxic membrane binding anticancer drug, induces glioma cell differentiation and autophagy. Proc Natl Acad Sci USA 109:8489–94. 22586083 10.1073/pnas.1118349109 PMC3365159

[CIT0033] Ugurel S, Röhmel J, Ascierto PA, et al. (2016). Survival of patients with advanced metastatic melanoma: the impact of novel therapies. Eur J Cancer 53:125–34. 26707829 10.1016/j.ejca.2015.09.013

[CIT0034] Wrobel D, Ionov M, Gardikis K, et al. (2011). Interactions of phosphorus-containing dendrimers with liposomes. Biochim Biophys Acta 1811:221–6. 21129500 10.1016/j.bbalip.2010.11.007

[CIT0035] Zhao M, Sano D, Pickering CR, et al. (2011). Assembly and initial characterization of a panel of 85 genomically validated cell lines from diverse head and neck tumor sites. Clin Cancer Res 17:7248–64. 21868764 10.1158/1078-0432.CCR-11-0690 PMC3229662

